# Magnetophoretic Sorting of Single Cell-Containing Microdroplets

**DOI:** 10.3390/mi7040056

**Published:** 2016-03-30

**Authors:** Younggeun Jo, Fengshan Shen, Young Ki Hahn, Ji-Ho Park, Je-Kyun Park

**Affiliations:** 1 Department of Bio and Brain Engineering, Korea Advanced Institute of Science and Technology (KAIST), 291 Daehak-ro, Yuseong-gu, Daejeon 34141, Republic of Korea; ygjo@kaist.ac.kr (Y.J.); bongsun@kaist.ac.kr (F.S.); jihopark@kaist.ac.kr (J.-H.P.); 2 Samsung Electronics, 4 Seocho-daero 74-gil, Seocho-gu, Seoul 06620, Republic of Korea; hahnv79@gmail.com

**Keywords:** magnetic nanoparticles, magnetophoresis, separation, single cell, *Thalassiosira eccentrica*

## Abstract

Droplet microfluidics is a promising tool for single-cell analysis since single cell can be comparted inside a tiny volume. However, droplet encapsulation of single cells still remains a challenging issue due to the low ratio of droplets containing single cells. Here, we introduce a simple and robust single cell sorting platform based on a magnetophoretic method using monodisperse magnetic nanoparticles (MNPs) and droplet microfluidics with >94% purity. There is an approximately equal amount of MNPs in the same-sized droplet, which has the same magnetic force under the magnetic field. However, the droplets containing single cells have a reduced number of MNPs, as much as the volume of the cell inside the droplet, resulting in a low magnetic force. Based on this simple principle, this platform enables the separation of single cell-encapsulated droplets from the droplets with no cells. Additionally, this device uses only a permanent magnet without any complex additional apparatus; hence, this new platform can be integrated into a single cell analysis system considering its effectiveness and convenience.

## 1. Introduction

Single cell sorting is defined as the compartmentalization of a heterogeneous mixture of cells and single cell analysis is of unique importance in cell biology, immunology, and genetics as well as cellular diagnostics [1,2,3]. As a major tool for cell sorting, flow cytometry, especially fluorescence-activated cell sorters, is widely exploited for high-speed cell sorting under an applied electric field after selectively charging objects of interest [4]. To overcome limitations in flow cytometry such as expensive equipment and complexities, however, several studies for single-cell analysis and sorting were carried out using microfluidics [5,6]. In this context, droplet microfluidics has been regarded as a promising tool for the study of cells at the single unit level [7,8]. Droplets provide a proper microenvironment for single cell study by isolation of desired cells inside a tiny volume of the droplets and enable the high throughput screening and analysis of single cells, including mammalian cells [9,10] and bacteria [11,12]. Recently, droplet-based microfluidics using alginate hydrogel microcapsules has been used for screening of microalgal cells based on their lipid content at the single cell level [13,14].

Droplet microfluidics for the encapsulation of single cells still remains a challenging issue caused by a low ratio of droplets containing single cells. The number of cells encapsulated in a droplet is dependent on the cell concentration in the aqueous phase, following the Poisson statistics [9]. To enhance the fraction of single-cell droplets, some efforts such as hydrodynamic self-organizing [15] and close-packed ordering [16] of particles or cells have been reported. However, these methods were only demonstrated with spherical mammalian cells, which are relatively uniform size and shape compared to the microalgae. Microalgal species vary considerably in size and shape. Depending on the species, they range from a few micrometers to a few tens of micrometers [17]. To overcome the limits of existing hydrodynamic methods, adequate droplet manipulation should be needed for a high ratio of single-cell droplets. To do this, droplet sorting approaches based on various principles such as dielectrophoretic force [18], pneumatic pressure [19], surface acoustic wave [20], and microchannel geometry [21] have been developed. However, these trials have limitations requiring additional complex equipment and result in the heterogeneous size distribution of droplets.

Herein, we present a magnetophoretic sorting method based on the relative magnetization of single cell-containing droplets, in which magnetic nanoparticles (MNPs) are included. The same-sized droplets contain approximately equal amounts of MNPs, showing the same degree of magnetization under the constant magnetic field in a microchannel. However, the number of MNPs inside the droplets containing single cells is reduced by the volume of the single cell, resulting in low magnetization. To sort out the single cell-encapsulated droplets, we exploit this different magnetic force between single cell-encapsulated droplet and empty droplet, caused by the change of the number of MNPs inside the droplet.

## 2. Materials and Methods

### 2.1. Materials

*Thalassiosira eccentrica* and their culture media (f/2) [22,23] were obtained from the Korean Marine Microalgae Culture Center (Busan, Korea). The shape of microalgae is cylindrical and their average diameter is 39.9 μm (±3.1 μm). Polystyrene microbeads with a diameter of 45 μm were purchased from Polysciences, Inc. (Warrington, PA, USA). Neutral dextran-coated magnetic nanoparticles with a diameter of 56 nm or less were kindly provided from Professor Ji-Ho Park (KAIST, Korea) [24]. A silicon oil was purchased from Sigma-Aldrich (St. Louis, MO, USA). A permanent magnet (NdFeB35, *w* × *l* × *h* = 25 mm × 50 mm × 10 mm) was bought from Magtopia Co. (Gumi, Korea). All fluids were injected via syringe pumps that were purchased from Harvard Apparatus (Holliston, MA, USA).

### 2.2. Cultivation of Microalgae

*Thalassiosira eccentrica* (KMMCC-666) was cultured and maintained at a temperature of 20 ± 1 °C in f/2 media for 3 weeks before the experiment. They were incubated under constant shaking with an agitation speed of 130 rpm and continuous illumination with a 3000-lux intensity lamp.

### 2.3. Device Fabrication

This device consisted of a PDMS microchannel and a nickel microstructure. The PDMS microchannel was fabricated by standard soft lithographic technology. Briefly, a negative photoresist, SU-8 2025 (MicroChem Corp., Newton, MA, USA), was spin-coated (1500 rpm) on a Si wafer to reach a final thickness of 50 μm. After pre-baking for 3 min at 65 °C and 7 min at 95 °C, the photoresist was exposed to UV light (160 mJ/cm^2^) via a photo mask for patterning the desired microchannel. After post-baking for 1 min at 65 °C and 6 min at 95 °C, the UV-exposed photoresist was developed for 5 min. This SU-8 master mold was used to prepare the PDMS microchannel. PDMS base monomer and curing agent (Sylgard 184, Dow Corning, Midland, MI, USA) were mixed at a 10:1 ratio and then poured on the SU-8 master mold. After baking for 50 min at 90 °C, the PDMS device was peeled off the master.

The nickel microstructure was prepared by a nickel electroplating method. For nickel electroplating, chrome and gold seed layers were deposited on glass wafers by metal sputtering, and negative photoresist (thickness of 150 μm), THB 151N, was then patterned on the seed layers by standard photolithography. After nickel electroplating, a chemical mechanical polishing was followed to obtain a final thickness of 100 μm. The photoresist was developed using a JSR THB-S1 stripper (JSR Corp., Tokyo, Japan) for about 2 h at 60 °C. The nickel microstructure (*w* × *l* × *h* = 2 mm × 10 mm × 100 μm) was detached and aligned on the PDMS device to be located nearby the microchannel. Then, the PDMS devices were exposed to an oxygen plasma for 1 min to bond with glass slide. For the hydrophobic surface of the microchannel, the PDMS devices were incubated for 3 days at 65 °C before experiments.

## 3. Results and Discussion

### 3.1. Design Theory for Magnetophoretic Separation of Single Cell-Containing Microdroplet

Considering an aqueous droplet containing MNPs under an inhomogeneous magnetic field, the magnetophoretic movement of MNPs allows the droplets to move toward a higher magnetic field gradient with the help of the interfacial tension of droplets. The magnetic force (***F****_m_*) [25] acting on each MNP inside a droplet is given by Equation (1):

(1) $$F_{m}=(V_{m}\Delta \chi \nabla B^{2})/(2\mu_{0})$$

where *V_m_* is the volume of a MNP, ∆χ is the net magnetic susceptibility of magnetic nanoparticle in aqueous solution (m^3^·kg^−1^), ∇***B*** is the gradient of magnetic flux density (T/m), and μ_0_ is the vacuum permeability (N/A^2^). The total magnetic force (***F****_tm_*) of MNPs inside a droplet is the sum of the magnetic force acting on each MNP inside the droplet.

(2) $$F_{tm}=N_{m}F_{m}=(2\pi R_{m}{}^{3}N_{m}\Delta \chi \nabla B^{2})/(3\mu_{0})$$

where *N_m_* is the number of MNPs inside a droplet and *R_m_* is the radius of single MNP. This total magnetic force allows the droplets to move toward a higher magnetic field gradient. Stokes’ drag force (***F****_D_*) by the magnetophoretic movement of droplets is represented by the following Equation (3):

(3) $$F_{D}=-6\pi R_{d}\eta v$$

(4) $$F_{tm}=-F_{D}$$

where ***v*** is the velocity of the droplet, *R_d_* is the radius of droplet, and η is the viscosity of the aqueous medium. Considering Equations (2)–(4), the velocity of the droplets (***v***) is represented by the following Equation (5):

(5) $$v=(N_{m}R_{m}{}^{3}\Delta \chi \nabla B^{2})/(9R_{d}{\eta \mu }_{0})$$

Accordingly, the lateral deflection (*L*) of droplets at the end of the separation channel can be represented by the product of the velocity of droplets and exposure time (*t*), for which the droplets can pass through the separation region under the magnetic field. Equation (5) shows that the lateral velocity of MNP-containing droplets could be controlled by an adjustment of the number of MNPs inside the droplet. Co-encapsulation of monodisperse MNPs and cells into a droplet reduces the number of MNPs inside the droplet because of the occupation of cells within the same sized droplet. Considering the single cell-encapsulated droplet, the number of MNPs inside the droplet, *N_s_*, is reduced by as much as the cell volume:

(6) $$N_{s}=N_{e}\left\{1-(R_{cell}/R_{d}{)}^{3}\right\}$$

where *N_e_* is the number of MNPs inside an empty droplet, which does not contain a cell, and *R_cell_* is the radius of the encapsulated cells. Consequently, the difference of lateral deflection (Δ*L*) is caused by the difference of the velocity of single cell-encapsulated droplets and empty droplets:

(7) $$\Delta L/L=(N_{e}-N_{s})/N_{m}$$

**Figure 1:**
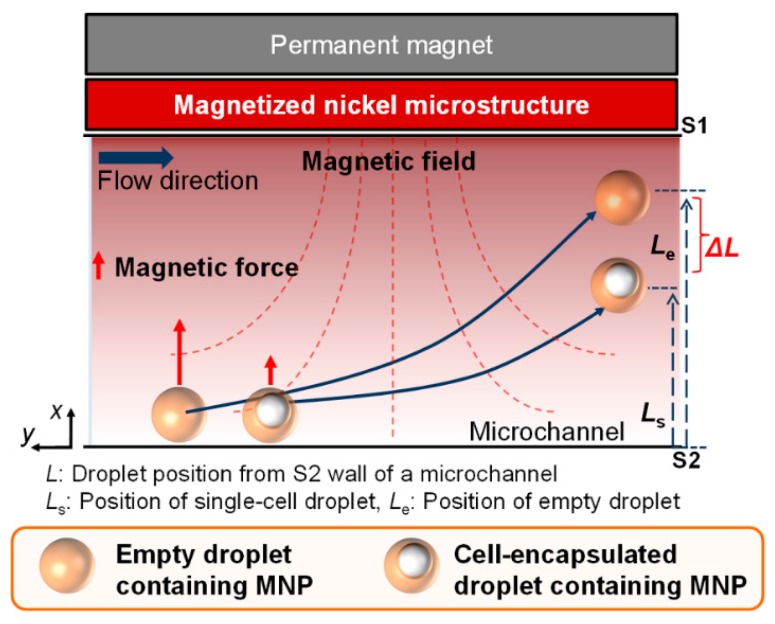
Principle of magnetophoretic sorting of single-cell droplet. Magnetic force acting on single-cell encapsulated droplets is less than that acting on empty droplets. *L* is lateral displacement of a droplet from the bottom side wall (S2) of the microchannel.

If this difference of lateral deflection between single cell-encapsulated droplets and empty droplets is enough, it is possible to sort one type of droplet among the mixed droplets (Figure 1). In addition, lateral deflection is also affected by the initial concentration of MNPs that are injected into the inlet and the relative difference of volume between single cell-encapsulated droplets and empty droplets.

**Figure 2:**
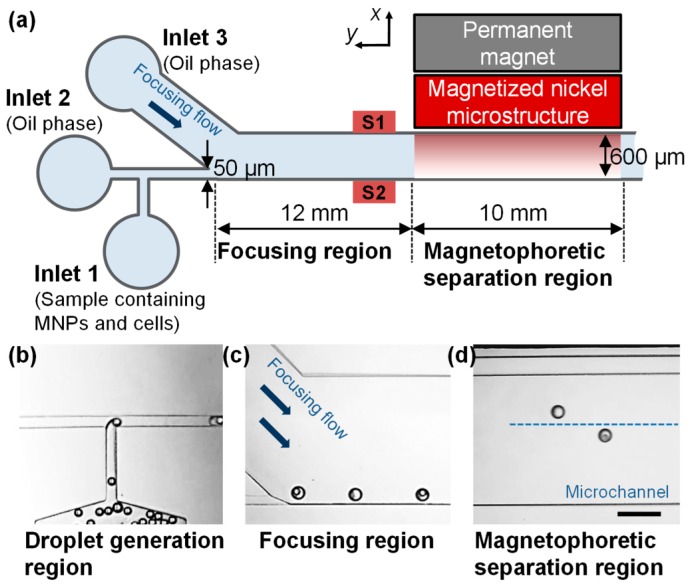
(**a**) Schematic view of a microfluidic device for magnetophoretic droplet sorting; (**b**) Droplets are generated with MNP solution and cells (or microbeads); (**c**) The droplets are focused to the side wall (S2) of the microchannel by oil phase flow from inlet 3; (**d**) Single cell (or microbead)-encapsulated droplet are separated by magnetic field. The scale bar is 200 μm.

**Figure 3:**
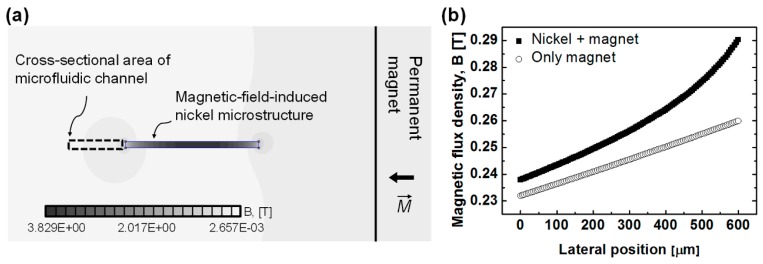
(**a**) Numerical analysis of the enhanced magnetic flux density at the cross section of the microchannel to evaluate the effect of a nickel microstructure (solved by the finite element method magnetics (FEMM)); (**b**) Comparison between the magnetic flux densities according to the presence of a nickel microstructure.

A simple and robust microfluidic device for magnetophoretic sorting of single cells is composed of a permanent magnet and a microchannel that is fabricated by poly(dimethylsiloxane) (PDMS). Figure 2a shows a schematic view of microfluidic device for magnetophoretic droplet sorting. The microchannel is composed of three main regions: a droplet generation region, a focusing region, and a magnetophoretic separation region. In the droplet generation region, droplets were generated via a T-shaped microchannel with a width of 50 μm and height of 50 μm. The droplets were generated with MNP solution, and the average diameter of the droplets was measured to be 69.9 μm (±1.1 μm) (Figure 2b). The droplets generated at the T-shaped microchannel were focused to the side wall (S2) of the microchannel by the oil phase flow from inlet 3 in the focusing region (Figure 2c). The focused droplets are then laterally deflected by attractive magnetic force in the magnetophoretic separation region (Figure 2d). The length of focusing and separation regions was 12 and 10 mm, respectively. The height of a microchannel was designed to be higher than the diameter of droplets to minimize resistance between droplets and microchannel walls. Lateral displacement of the droplets from wall S2 was measured 10 mm apart from the initial point of separation region. In order to enhance the magnetic field gradient across the microchannel, a nickel microstructure as a ferromagnetic material was embedded at the side of the separation region. The enhancement effect of magnetic field gradient is confirmed by a simulation with the finite element method magnetics (FEMM) (Figure 3).

### 3.2. Effect of MNP Concentrations inside the Droplets

**Figure 4:**
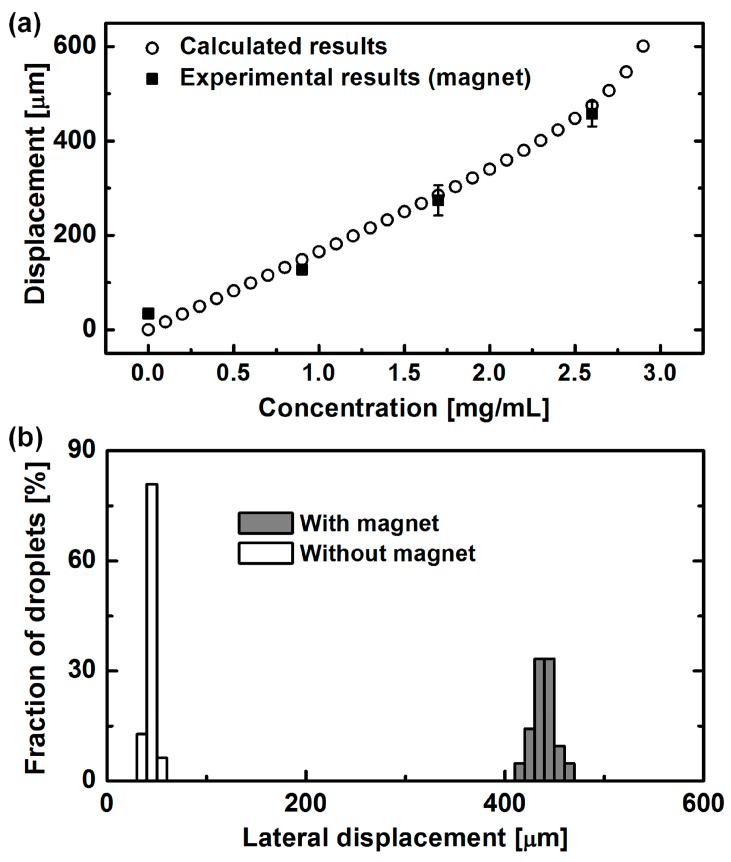
The effect of magnetic force acting on the droplets. (**a**) Lateral displacement of droplets according to the concentration of MNPs inside the droplets. Higher concentration of MNPs leads to the higher magnetophoretic deflection. (**b**) Lateral displacement of droplets with and without magnetic field. Droplets were laterally deflected only by magnetic force.

First, we confirmed the effect of MNP concentrations inside the droplets for effective magnetophoretic sorting. A magnetic force acting on the droplets is proportional to the number of MNPs inside the droplets. To estimate the suitable concentration of MNPs inside the droplets for the separation of single cells, lateral movement of droplets for the various concentrations of MNPs was calculated with Equation (5), in which the magnetic flux density was that of a permanent magnet enhanced by ferromagnetic material (simulated with FEMM). In this calculation, we used exposure times, for which the droplets can pass through the separation region under the magnetic field. This calculation was performed using MATLAB. Consequently, it is confirmed that the higher concentration of MNPs, which means a large number of MNPs inside the droplet, shows a larger displacement of the droplet by a magnetic force (Figure 4a, open circle). Based on these simulation results, we adjusted the appropriate concentrations of MNPs.

For selecting suitable concentration of MNPs (approximately 4.885 × 10^18^ particles/mg) inside the droplets, the droplets with four different concentrations of MNPs (0, 0.9, 1.73, and 2.6 mg/mL) were generated at the T-shaped microchannel. MNP solutions for four different concentrations were injected into inlet 1 with a flow rate of 3 μL/h, and mineral oil was injected into inlet 2 with a flow rate of 17 μL/h. For the focusing of the droplets generated, the flow rate of mineral oil from inlet 3 finally settled to 300 μL/h. Lateral displacement of the injected droplets was measured at the end of the separation region, which set the bottom side wall (S2) as zero point. Under the magnetic field, the calculated results obtained by Equation 5 were in good agreement with the experimental results, showing that lateral displacement of droplets increased according to the MNP concentrations (Figure 4a). While the droplets including no MNPs (0 mg/mL) were aligned at the bottom side (S2) under the external magnetic field, those with 0.9 mg/mL of MNPs were attracted toward the upper side (S1), maintaining the lateral displacement of 127 μm. The average lateral displacement of the droplets encapsulating MNPs of 1.73 mg/mL and 2.6 mg/mL was found to be 274 μm and 457 μm, respectively. Without a magnetic field, all droplets were expected to be aligned toward the bottom side wall (S2) regardless of the concentration of MNPs, which was confirmed with 2.6 mg/mL of the MNP solution (Figure 4b).

This result demonstrated the possibility of sorting the desired droplets according to the number of MNPs inside the droplets. As a result, we found that 2.6 mg/mL of MNPs was the proper concentration for the separation of the droplets in this experimental set-up. When the higher concentration of MNPs (>2.6 mg/mL) were employed, the droplets were completely attracted toward the upper side wall (S1), resulting in the difficulty of separating the desired droplets according to the number of MNPs inside the droplets.

### 3.3. Effect of Magnetophoretic Mobility by the Occupation of a Microbead inside the Droplets

**Figure 5:**
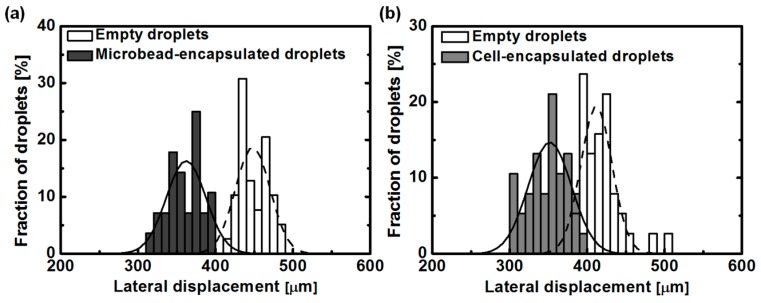
Results of magnetophoretic sorting of single cell (or microbead)-containing droplets (*n* = 450). (**a**) Distribution of lateral position of empty droplets and single microbead-encapsulated droplets; (**b**) Separation of single cell (*Thalassiosira eccentrica*)-encapsulated droplets.

On the basis of results for the change of MNP concentrations, we investigated the lateral deflection of droplets containing single microbeads. The same-sized droplets contain approximately equal amounts of MNPs, showing the same magnetic force acting on the droplets. An occupation of a microbead inside a droplet reduces the number of MNPs inside the droplet because MNPs are monodisperse in solution. The magnetic force acting on a microbead-encapsulated droplet decreases by the reduced amount of MNPs inside the droplet. This reduced magnetic force leads to the difference of lateral deflection between microbead-encapsulated droplets and empty droplets. To demonstrate this difference in lateral displacement, microbeads with a diameter of 45 μm and MNPs solution with a concentration of 2.6 mg/mL were used. Two different droplets (e.g., single microbead-encapsulated droplets and empty droplets) containing monodisperse MNPs were generated with the same size (70 μm-diameter droplets). The droplets generated from the T-shaped microchannel were focused and aligned at side wall S2 by the flow of mineral oil from inlet 3. While the droplets passed through the separation region, the difference of magnetic force acting on two types of droplets led to the separation of the droplets. Figure 5a shows the distribution of lateral displacement for the two types of droplets at the end of the separation region. It was confirmed that empty droplets were more attracted toward side wall S1 than single microbead-encapsulated droplets. The average lateral displacement of empty droplets and single microbead-encapsulated droplets was found to be 448 and 360 µm, respectively. It seems that a large distribution of single microbead-encapsulated droplets was caused by the size variation (CV ≤ 10%, provided from a manufacturer) of microbeads because the number of MNPs inside the droplet is dependent on the volume of encapsulated microbead. In this experiment, due to the physical size of two microbeads, it is very difficult to generate the droplets containing more than two microbeads at the experimental condition for the droplet generation.

### 3.4. Magnetophoretic Separation of Single Cell-Encapsulated Droplets

To demonstrate the separation of single cell-encapsulated droplets and empty droplets, a microalgae, *Thalassiosira eccentrica* was used as a target cell. To prevent the electrostatic adsorption [26,27] of magnetic nanoparticles by microalgae, we employed neutral dextran-coated magnetic nanoparticles [24]. Two types of droplets (single cell-encapsulated droplets and empty droplets) were generated with mineral oil as an oil phase and an f/2 media-based MNP solution as an aqueous phase in a T-shaped microchannel. For the effective encapsulation of cylindrical cells, the flow rates of inlets 1 and 2 were adjusted to be 6 and 34 μL/h, respectively, slightly faster than the flow rate in the above experiments using microbeads, keeping the ratio of flow rates between inlets 1 and 2 for generating the same-sized droplet. Generated droplets were focused toward the bottom side wall (S2) by focusing flow from inlet 3 (at a flow rate of 300 μL/h). Other conditions were the same as the experiment using microbeads. As a result, the fraction of single cell-encapsulated droplets was about 4.9%. This low proportion of single cell encapsulation was associated with the shape of *Thalassiosira eccentrica*, which is a cylindrical cell with diameters of 39.9 μm (±3.1 μm). It is hard to encapsulate a single cell into a droplet using only a hydrodynamic flow because angulated and large cells naturally settle at the bottom of a microchannel. For this reason, to encapsulate angulated and large microalgae such as *Thalassiosira eccentrica*, an adequate droplet manipulation technique is necessary.

Figure 5b shows the distribution in lateral displacement of two types of droplets. The average lateral displacement of empty droplets and single cell-encapsulated droplets was observed at 416 and 349 µm, respectively. Accordingly, the difference between single cell-encapsulated droplets and empty droplets is about 67 μm. Single cell-encapsulated droplets were less attracted because of the reduced number of MNPs inside the droplet, as much as the volume of single microalgae. Large variation in microalgae size caused a relatively broad distribution in the lateral positions of cell-encapsulated droplets. Single cell-encapsulated droplets could be separated from empty droplets with a separation efficiency of 94.87% by a bifurcated channel at a lateral position of 390 μm. This separation efficiency was calculated as the ratio of single cell-encapsulated droplets to whole droplets that passed through below the lateral position of 390 μm.

## 4. Conclusions

We successfully demonstrated a simple and robust magnetophoretic sorting method of single cell-encapsulated droplets from the empty droplets without cells. To sort a single cell effectively, a single cell was co-encapsulated with monodisperse magnetic nanoparticles into the droplets with a diameter of 70 μm. The reduced number of MNPs by the occupation of a single cell in a droplet caused a lower magnetic force compared to the droplets without single cells, resulting in a difference in the lateral positions of droplets. Consequently, we sorted single microalgae (*Thalassiosira eccentrica*)-containing droplets from the empty droplets with >94% purity. This device enables the separation of single cells using only a permanent magnet without any complex additional apparatus; therefore, this new platform can be integrated into a single cell analysis system for its effectiveness and convenience.
